# Supplement Use and Other Characteristics Among Pregnant Women with a Previous Pregnancy Affected by a Neural Tube Defect — United States, 1997–2009

**Published:** 2015-01-16

**Authors:** Annelise Arth, Sarah Tinker, Cynthia Moore, Mark Canfield, AJ Agopian, Jennita Reefhuis

**Affiliations:** 1 Division of Birth Defects and Developmental Disabilities, National Center on Birth Defects and Developmental Disabilities, CDC; 2 Texas Department of State Health Services; 3 University of Texas School of Public Health

Neural tube defects (NTDs) include anomalies of the brain (anencephaly and encephalocele) and spine (spina bifida). Even with ongoing mandatory folic acid fortification of enriched cereal grain products, the U.S. Preventive Services Task Force recommends that women of childbearing potential consume a daily supplement containing 400 *μ*g–800 *μ*g of folic acid (1). Women with a prior NTD-affected pregnancy have an increased risk for having another NTD-affected pregnancy, and if they are planning another pregnancy, the recommendation is that they consume high-dosage folic acid supplements (4.0 mg/day) beginning ≥4 weeks before conception and continuing through the first 12 weeks of pregnancy (2). To learn whether folic acid supplementation (from multivitamins or single- ingredient supplements) was commonly used during pregnancy by women with a previous NTD-affected pregnancy, supplement use was assessed among a convenience sample of women with a previous NTD-affected pregnancy who participated in the National Birth Defects Prevention Study (NBDPS), a case-control study of major birth defects in the United States. Characteristics of women who previously had an NTD-affected pregnancy and whose index pregnancy (pregnancy included in NBDPS) was either affected by an NTD (N = 17) (i.e., recurrence-cases) or resulted in a live-born infant without a major birth defect (N = 10) (i.e., recurrence-controls) were assessed. Taking a supplement that included folic acid was more common among recurrence-control mothers (80%) than recurrence-case mothers (35%). The recommendation that women should take folic acid supplements just before and during early pregnancy is not being followed by many women and offers an opportunity for NTD prevention, especially among women who are at a higher risk because they have had a previous pregnancy affected by an NTD.

Before folic acid fortification in the United States, the NTD recurrence risk was estimated to be about 2%–5% (3). Randomized controlled trials among women with a previous NTD-affected pregnancy demonstrated that a high-dosage folic acid supplement taken periconceptionally reduces the risk for recurrence up to 100% depending on background prevalence (2). Because a high dosage of folic acid (4.0 mg per day) recommended for women with a previous NTD-affected pregnancy exceeds the content of typical prenatal or other multivitamins (typically 400 *μ*g–800 *μ*g of folic acid), women at high risk might be prescribed dietary supplements with folic acid dosages >1.0 mg (4).

NBDPS is a multicenter case-control study conducted to assess risk factors for selected major birth defects. Cases were ascertained through population-based surveillance programs in 10 states (Arkansas, California, Georgia, Iowa, Massachusetts, New Jersey, New York, North Carolina, Texas, and Utah) and included live births, fetal deaths (in all sites except New Jersey), and pregnancy terminations (in all sites except Massachusetts and New Jersey) affected by at least one of the included major birth defects; cases with recognized syndromes or single-gene disorders were excluded. NBDPS includes only one eligible pregnancy per mother. Controls were live births with no major birth defects selected from the same geographically defined regions, identified through hospital logs or birth certificates. Pregnancies with an estimated date of delivery from October 1, 1997, through December 31, 2009, were included, and computer-assisted telephone interviews were conducted with women 6 weeks–24 months after their estimated date of delivery. Interview topics included pregnancy history, family history of birth defects, maternal health and medication use during pregnancy, and demographics.

The descriptive analysis was limited to mothers who reported a previous NTD-affected pregnancy during the interview. Recurrence-case mothers were those whose index pregnancy was affected by encephalocele, anencephaly, or spina bifida. Recurrence-control mothers were those who had a previous NTD-affected pregnancy, whose index pregnancy was included in NBDPS as a control. Although NBDPS is a population-based study, this sample of mothers with a previous NTD-affected pregnancy should be considered a convenience sample because the only mothers eligible to be included were those who did not participate in NBDPS with a previous NTD-affected pregnancy because the birth occurred before the study began, was outside the study area, or was not ascertained, or because the mother did not participate.

Frequencies of several maternal characteristics and pregnancy exposures among cases and controls were considered: year of estimated date of delivery, clinical characteristics of the index birth and previous NTD-affected pregnancy, maternal age, race/ethnicity, education, pre-pregnancy body mass index, vitamin and medication use, diabetes, and pregnancy intention. NBDPS does not collect information on folic acid dosage, and therefore the only indicator of whether a mother might have taken high-dosage folic acid supplement was reported use of a single-ingredient folic acid supplement, in which case it is possible that she took the recommended amount.

A previous NTD-affected pregnancy was reported by 27 mothers (Table). Of these, 17 had index pregnancies that were also NTD-affected (recurrence-cases), and 10 had index pregnancies that resulted in a live-born infant without a major birth defect (recurrence-controls). Six recurrence-case mothers (35%) reported taking either a single-ingredient folic acid supplement or a prenatal or multivitamin in the 3 months before conception, compared with eight recurrence-control mothers (80%). Reported use of a single-ingredient folic acid supplement was more common among recurrence-control mothers, 70% (seven of 10), compared with 18% (three of 17) of recurrence-case mothers.

Over one third of recurrence-case mothers were Hispanic (six of 17), whereas only one of the 10 recurrence-control mothers was Hispanic. No Hispanic or non-Hispanic black case mothers reported using a supplement with folic acid, whereas 83% of non-Hispanic white case mothers and 88% of non-Hispanic white control mothers did.

Intending to become pregnant at the time of conception was reported by most case (10 of 17) and control (eight of 10) mothers. However, only half of case mothers intending pregnancy (five of 10) took a folic acid supplement during the preconception period. Single-ingredient folic acid supplement use was reported among only two of 10 case and six of eight control mothers intending pregnancy.

What is already known on this topic?

Women who have had a previous pregnancy affected by a neural tube defect (NTD) are at an increased risk for having another NTD-affected pregnancy. The daily use of a high-dosage (4.0 mg) folic acid supplement from ≥4 weeks before through the first 12 weeks of pregnancy has been shown to decrease risk for having a subsequent NTD-affected pregnancy.

What is added by this report?

Among 17 mothers with an NTD-affected pregnancy enrolled in the National Birth Defects Prevention Study and a history of a previous NTD-affected pregnancy, 35% reported taking a folic acid supplement, whereas among 10 mothers of live-born infants without a birth defect who had a previous NTD-affected pregnancy (i.e., controls), 80% reported taking a folic acid supplement. Six of 17 mothers with a second NTD-affected pregnancy were Hispanic, whereas only one of 10 control (second pregnancy was not NTD-affected) mothers was Hispanic; none of the seven Hispanic mothers reported using a single-ingredient folic acid supplement.

What are the implications for public health practice?

Many women who have had an NTD-affected pregnancy and are planning a subsequent pregnancy do not take a folic acid supplement. Clinicians and local health departments need to be aware that women at higher risk for having an NTD-affected pregnancy might not be following current folic acid recommendations and need to tailor prevention messages to encourage use.

Over one third (six of 17) of recurrence-case mothers were obese (body mass index ≥ 30) compared with one of 10 recurrence-control mothers (10%). Two recurrence-case mothers reported use of opioid medication; one in the 2 months before pregnancy and one during the 3 months before and after the start of pregnancy. One of the three recurrence-case mothers who reported use of a single-ingredient folic acid supplement stated use of an opioid; a second mother had type 2 diabetes and reported taking metformin.

## Discussion

Taking a folic acid supplement (single vitamin or as part of a multivitamin) was reported by a low percentage of NBDPS recurrence-case mothers. Although the sample was small, the results are consistent with a protective effect of folic acid against recurrent NTDs, because recurrence-control mothers more often reported preconception use of folic acid supplements than recurrence-case mothers. Preventing the recurrence of NTDs by managing maternal risk factors before conception, including folic acid intake and obesity, presents important opportunities for public health.

A racial/ethnic disparity in preconception use of a high-dosage folic acid supplement has been observed previously; a Texas-based study of 195 mothers at high risk for NTD recurrence revealed a significant difference in folic acid–containing supplement use between non-Hispanic white (64.7%) and Hispanic (16.5%) mothers, even though recall of receiving postpartum advice did not vary (5). However, mothers who recalled receiving advice were more likely to take supplements than those who did not (5). Importantly, active outreach to mothers with a previous NTD-affected pregnancy has been shown to increase folic acid supplement use among mothers in South Carolina and along the Texas-Mexico border (6,7).

Prepregnancy obesity has been associated with NTD risk, and obesity was more common among recurrence-case mothers than recurrence-control mothers (8). The data do not allow for the identification of causative factors for these recurrent NTD cases, but it is of note that two of three recurrence-case mothers who reported taking a single-ingredient folic acid supplement reported other NTD risk factors, specifically obesity, prepregnancy diabetes, and opioid medication use (8,9).

The findings in this report are subject to at least two limitations. First, because of small numbers and the study’s design, no statistical tests were performed, and recurrence risk could not be estimated. Second, for study years 1997–2005, the family history question did not distinguish between older and younger siblings, making it theoretically possible that some cases were classified as recurrent that were actually the first NTD-affected pregnancy for that mother. However, most NTD-affected siblings are older because the interview was performed as soon as possible after the estimated date of delivery of the pregnancy, on average 9 months.

This study suggests that awareness of the importance of folic acid supplement use in preconception and early pregnancy should be increased among women with a previous NTD-affected pregnancy. Barriers to implementing NTD recurrence prevention recommendations need to be identified and overcome. Communication of the importance of this preventive action by health care providers to women of childbearing age, especially those at high risk for another NTD-affected pregnancy, should be improved. Active outreach programs have been shown to be cost-effective in preventing NTD recurrences and might be considered (10).

## Figures and Tables

**TABLE:** Convenience sample of women with a previous pregnancy affected by a neural tube defect (NTD) — National Birth Defects Prevention Study, 1997–2009

Case no.	Case/Control	Previous pregnancy NTD	Index pregnancy NTD	Single-ingredient folic acid vitamin use*	Prenatal or multi-vitamin use*	Maternal race/ethnicity†	BMI category§	Medication summary B1–P2¶	Pregnancy intent summary
1	Case	SB	SB	Yes	No	White	Overweight	Opioid (morphine B2–B1, acetaminophen/hydrocodone B1); Abx (cephalexin B1 and P2–P5)	Did not care
2	Case	AN	AN	Yes	No	White	Obese	Ace-inhibitor (lisinopril B3–P1); diabetes oral medication (NOS B1–P1, metformin B3–P1)	Wanted to be pregnant then
3	Case	SB	SB	Yes	No	Other	Normal weight		Wanted to be pregnant then
4	Case	SB	SB	No	Yes	White	Overweight	Abx (antibiotic NOS B1)	Wanted to be pregnant then
5	Case	SB	SB	No	Yes	White	Obese	Opioid (acetaminophen/propoxyphene B3–P3)	Wanted to be pregnant then
6	Case	SB	SB	No	Yes	White	Normal weight		Wanted to be pregnant then
7	Case	SB	SB	No	No	Hispanic	Normal weight		Wanted to be pregnant then
8	Case	SB	SB	No	No	Hispanic	Obese		Got pregnant while consistently using contraception
9	Case	EN	AN	No	No	Hispanic	Overweight		Wanted to be pregnant then
10	Case	SB	SB	No	No	Hispanic	Obese		Wanted to wait until later
11	Case	AN	SB	No	No	Hispanic	Normal weight		Wanted to be pregnant then
12	Case	SB	AN	No	No	Hispanic	Obese		Wanted to wait until later
13	Case	SB	EN	No	No	Black	Overweight		Wanted to be pregnant then
14	Case	SB	SB	No	No	Black	Obese		Did not want to become pregnant at all
15	Case	SB	SB	No	No	Black	Overweight	Abx (amoxicillin/clavulanate P2)	Did not want to become pregnant at all
16	Case	AN	SB	No	No	White	Normal weight		Wanted to wait until later
17	Case	EN	EN	No	No	Other	Normal weight		Wanted to be pregnant then
18	Control	AN	CO	Yes	Yes	White	Normal weight	Abx (ciprofloxacin B1)	Wanted to be pregnant then
19	Control	SB	CO	Yes	Yes	White	Normal weight		Wanted to be pregnant then
20	Control	U	CO	Yes	Yes	White	Overweight		Wanted to wait until later
21	Control	AN	CO	Yes	Yes	White	Normal weight		Wanted to be pregnant then
22	Control	SB	CO	Yes	Yes	White	Obese	Opioid (cough syrup with codeine NOS P2)	Wanted to be pregnant then
23	Control	EN	CO	Yes	No	White	Normal weight		Wanted to be pregnant then
24	Control	AN	CO	Yes	No	White	Normal weight		Stopped using contraception to get pregnant
25	Control	SB	CO	No	Yes	Hispanic	Missing		Wanted to be pregnant then
26	Control	AN and SB	CO	No	No	White	Overweight		Got pregnant while consistently using contraception
27	Control	SB	CO	No	No	Other	Underweight	Abx (cephalexin B1–P7)	Wanted to be pregnant then

**Abbreviations:** BMI = body mass index; SB = spina bifida; AN = anencephaly; EN = encephalocele; U = unknown phenotype; CO = control; B3, B2, B1 = 3rd, 2nd, 1st month before pregnancy; P1, P2, and P3...P9 = 1st, 2nd, 3rd...9th month of pregnancy; Abx = antibiotic; NOS = not otherwise specified.

* B3–B1 self-reported use.

† Mothers of white, black, or other race are all non-Hispanic.

§ Units in kg/m^2^ where underweight = <18.5; normal weight = 18.5–24.9; overweight = 25.0–29.9; and obese = ≥30.0.

¶ B1–P2 self-reported use.
